# Relaxation dynamics in magnetic antidot lattice arrays of Co/Pt with perpendicular anisotropy

**DOI:** 10.1038/s41598-018-29903-8

**Published:** 2018-08-03

**Authors:** Sougata Mallick, Swapna Sindhu Mishra, Subhankar Bedanta

**Affiliations:** Laboratory for Nanomagnetism and Magnetic Materials (LNMM), School of Physical Sciences, National Institute of Science Education and Research (NISER), HBNI, Jatni, 752050 India

## Abstract

The topic of magnetic antidot lattice (MAL) arrays has drawn attention from both fundamental research as well as from application point of view. MAL arrays are promising candidates for making domain engineering in thin films. For various applications it is necessary to understand the magnetization reversal mechanism as well as the relaxation dynamics. In this context we have studied magnetic antidot lattice (MAL) arrays of Co/Pt with perpendicular anisotropy fabricated by combination of photolithography and sputtering deposition. Kerr microscopy domain imaging for the continuous thin film reveals the formation of typical bubble domains of perpendicular media with high anisotropy. However, presence of periodic holes in the MAL arrays lead to nucleation of localised smaller bubbles. We have performed simulations using object oriented micromagnetic framework (OOMMF) which reproduced the experimental results even considering antidot arrays in nano dimension. In literature it has been reported that in MAL arrays with in-plane anisotropy the domain propagation gets significantly hindered by the presence of the holes. However here we show that in perpendicularly magnetized Co/Pt the propagation of the domain walls is not restricted by the presence of the antidots. Further we have performed magnetic relaxation study and found that the global relaxation time for the MAL arrays of Co/Pt is faster as compared to it’s parent thin film. This behavior is opposite to what has been observed in literature for in-plane magnetized MAL arrays.

## Introduction

Fast switching of the magnetization in thin films and nanostructures is of utmost importance in high frequency inductors, microwave filters, spin valve, MRAM based devices, etc.^1,2^. Media writability and data retention demands complete understanding of the magnetization reversal and relaxation in patterned nanostructures^3^. Lithography patterned magnetic antidot lattice (MAL) arrays are receiving significant research interest over last two decades because of several factors, viz. dimension not being restricted by the superparamagnetic limit^4^ to the bit size, feasibility of tuning the spin waves as magneto-photonic crystals^5^, etc. Most of the works on the MALs so far have been focused on the in-plane magnetized systems where the lateral movement of the domain walls are engineered by incorporating the periodic holes^6–9^. The presence of these periodic holes in such in-plane magnetized MAL arrays hinders the path of propagation of the domains in lateral direction which essentially slows down the magnetic relaxation mechanism^9,10^. Our previous work about relaxation behavior in Co in-plane magnetized systems reveal that the relaxation time increases from 4.09 s in continuous thin film to 34.05 s of micro-dimensional triangular antidots^10,11^. The antidot edges pin the magnetic domains and hence more energy is required to complete the reversal. Further, lowering the dimension of the holes as well as the inter-separation lead to magnetic hardening^12,13^. However this magnetic hardening is usually accompanied by slower relaxation time in case of the MAL arrays^10^. The latter is not desirable for storage applications where rapid reversal of the spins is essential in reading and writing information^2,14^. In this context magnetic thin films with perpendicular magnetic anisotropy (PMA) exhibit the potential to overcome these limitations. In PMA based MAL systems the magnetic domain walls (Bloch walls) propagate in out-of-plane direction and hence is expected to face less hindrance in the path of propagation. In addition the thermal stability of the magnetization even at very low dimension is ensured by use of such materials with high PMA^15^. So far, a very few literature exist on MAL arrays with PMA which report the study of the magnetization reversal vis-a-vis lattice symmetry and dimension^16–23^, spin wave dispersion in magnonic crystals^24^, exchange bias^25^, etc. However, in literature there has been no report on magnetic relaxation study by introduction of periodic holes in MAL arrays having PMA.

In this paper, we report the investigation of magnetic domains and relaxation by state-of-art magneto-optic Kerr effect (MOKE) based microscopy in micro-dimensional MAL arrays of Co/Pt with PMA. The paper is organized in the following way. First the structural characterization of the MAL arrays has been performed by scanning electron microscopy. Afterwards static hysteresis loops have been measured for both the continuous thin films and the MAL arrays which indicate magnetic hardening for the latter case. Domains observed in case of MAL arrays although are locally nucleated by the periodic defects, however the domain propagation is not much hindered by the antidots. We demonstrate by simulation using object oriented micro magnetic framework (OOMMF) that the experimental observations can be reproduced and extended for MAL arrays consisting of holes with dimensions of few tens of nanometre. In order to gain insights on the effect of MAL arrays on the magnetic switching behavior we have performed relaxation experiments by Kerr microscopy for both the continuous thin films and MAL arrays. We observe that the relaxation in such antidots is faster in comparison to the parent continuous thin films. We discuss the relaxation behavior for the MAL arrays of PMA based Co/Pt to their counterpart with in-plane magnetic anisotropy reported in literature.

## Results and Discussion

**Figure 1:**
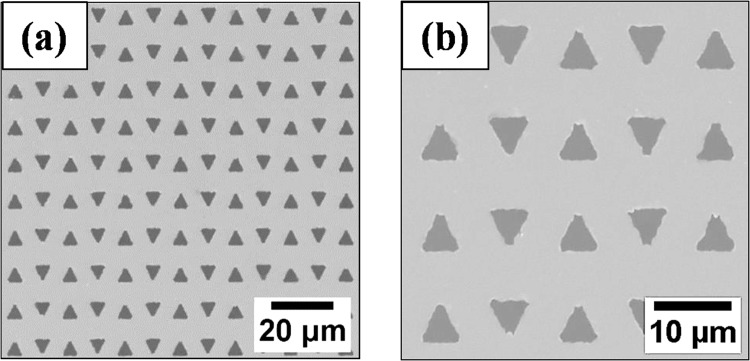
(**a**) SEM image of the triangular antidot (ES2), (**b**) zoomed-in view of the image shown in (**a**). The feature sizes and inter-separations between the holes are given in Table 1.

Figure 1(a) shows the SEM image for the triangular antidot (sample ES2). It is observed that the antidot pattern is uniform over the entire sample area. Figure 1(b) shows the zoomed-in view of the SEM image. Here the side length (*d*) of the (equilateral) triangular shaped holes is ~4.8 *μ*m. The centre-to-centre distance along the vertical and horizontal denominated by *D*_1_ and *D*_2_ are ~11.9 and 9.5 *μ*m, respectively.

**Figure 2:**
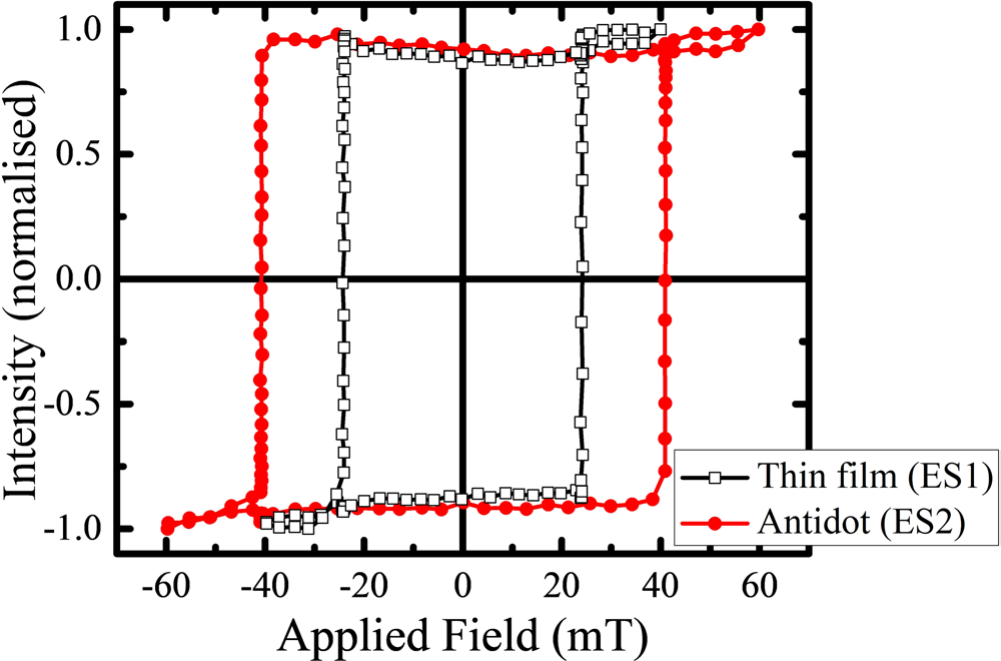
Hysteresis loops measured by Kerr microscope in polar mode for sample ES1 (black hollow square with line) and ES2 (red solid circle with line).

Figure 2 shows the hysteresis loops measured by Kerr microscope in polar mode for the continuous thin film sample ES1 (black hollow square) and the antidot sample ES2 (red solid circle). The extracted values of the coercive fields (*H*_*C*_) are 24.23 and 40.79 mT for ES1 and ES2, respectively. Therefore the periodic holes in the antidot sample lead to magnetic hardening. The periodic holes work as the nucleation centres and act as pinning barriers for the magnetic domains. To overcome the additional pinning, the reversal field increases for the MAL arrays. This in principle is a general behavior for MAL arrays irrespective of their anisotropy and architecture^9,10,12,13^. However the shape of the loops (shown in Fig. 2) indicate that the nature of magnetization reversal is same in both the cases.

**Figure 3:**
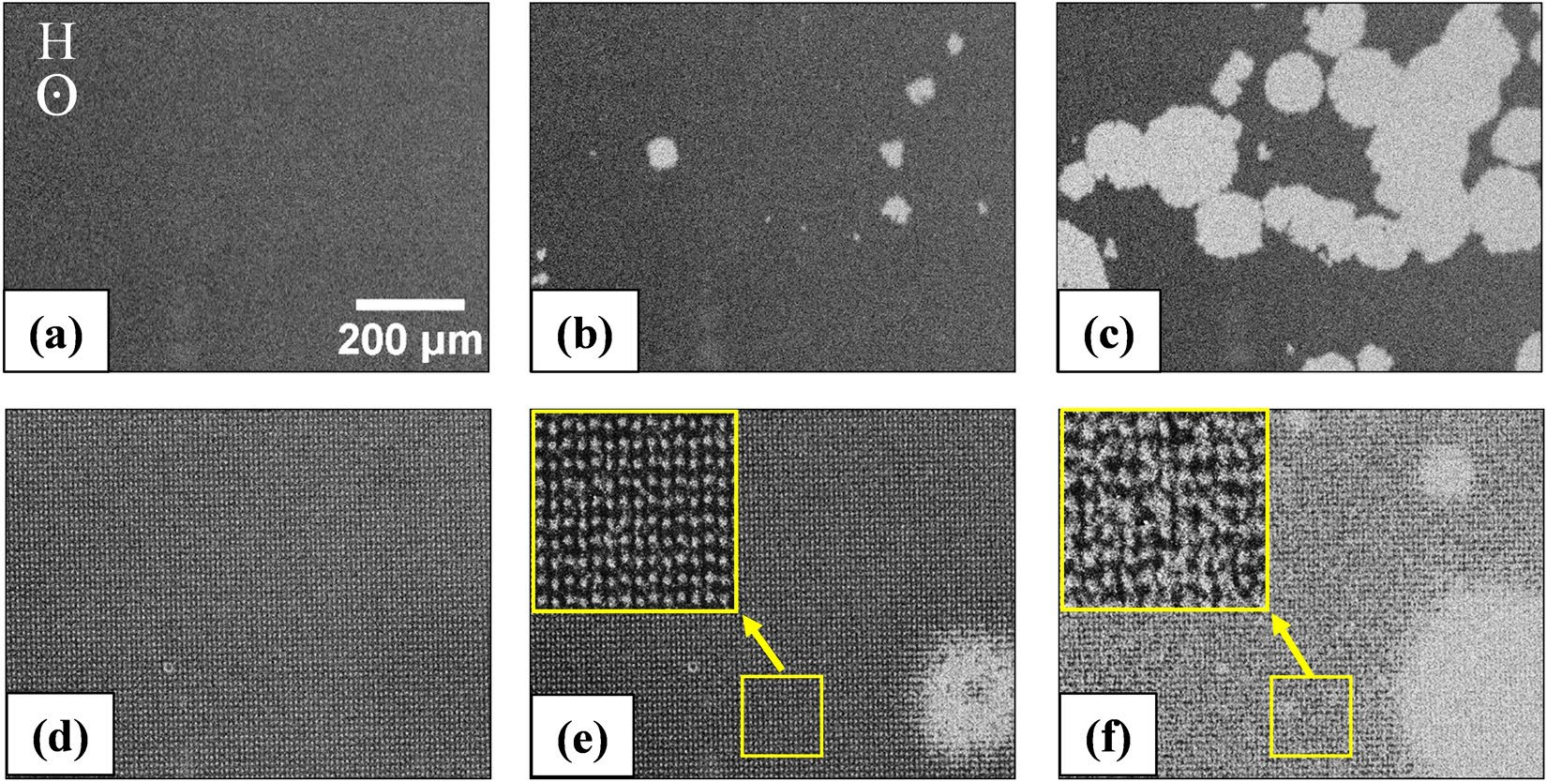
(**a**–**c**) and (**d**–**f**) are the domain images at -*H*_*S*_, *H*_*N*_, and *H*_*C*_, for samples ES1 and ES2, respectively. The direction of applied magnetic field and scale bar for all the images are same and shown in (**a**). The insets of (**e**) and (**f**) show the high-resolution images marked by the yellow boxes in the corresponding images.

Figure 3(a–c) show the domain images observed by Kerr microscopy for sample ES1 at -*H*_*S*_ (saturation field), *H*_*N*_ (nucleation field), and *H*_*C*_, respectively. It is observed that at *H*_*N*_ = 24.1 mT, bubble domains start nucleating (Fig. 3(b)). With increase of magnetic field the bubbles expand (Fig. 3(c)) before coalescing with each other to complete the reversal. It is known that uniform bubble domains are observed in thin films with *Q*(=*K*_*u*_/*K*_*d*_) $\gg$ 1, where *K*_*u*_ and *K*_*d*_ are the perpendicular anisotropy and stray field energy densities, respectively^26^. The individual bubble domains remain stable between the bubble collapse field (*h*_*bc*_) and the bubble strip out field (*h*_*bs*_) as discussed in ref.^26^. Figure 3(d–f) show the domain images for sample ES2 at -*H*_*S*_, *H*_*N*_, and *H*_*C*_, respectively. The sharp edges of the triangular holes act as pinning and nucleation centres for the bubble domains. This leads to nucleation of small localised bubbles in sample ES2 (inset of Fig. 3(e)). The domains are irregular in shape due to the distribution of energy landscapes in the antidot array. Increase of applied magnetic field leads to further nucleation of domains along with enhancement of size (Fig. 3(f)). The domain nucleation and propagation for samples ES1 and ES2 can be observed in supplementary videos 1 and 2, respectively. It is observed that although in case of the Co/Pt MAL arrays with PMA the reversal is nucleation dominated, however, the overall nature of the bubble domains is similar to its parent continuous thin film. It should be noted that, this behavior is different when comparing the domains of MAL arrays having in-plane anisotropy to their parent thin films^9,10^.

**Figure 4:**
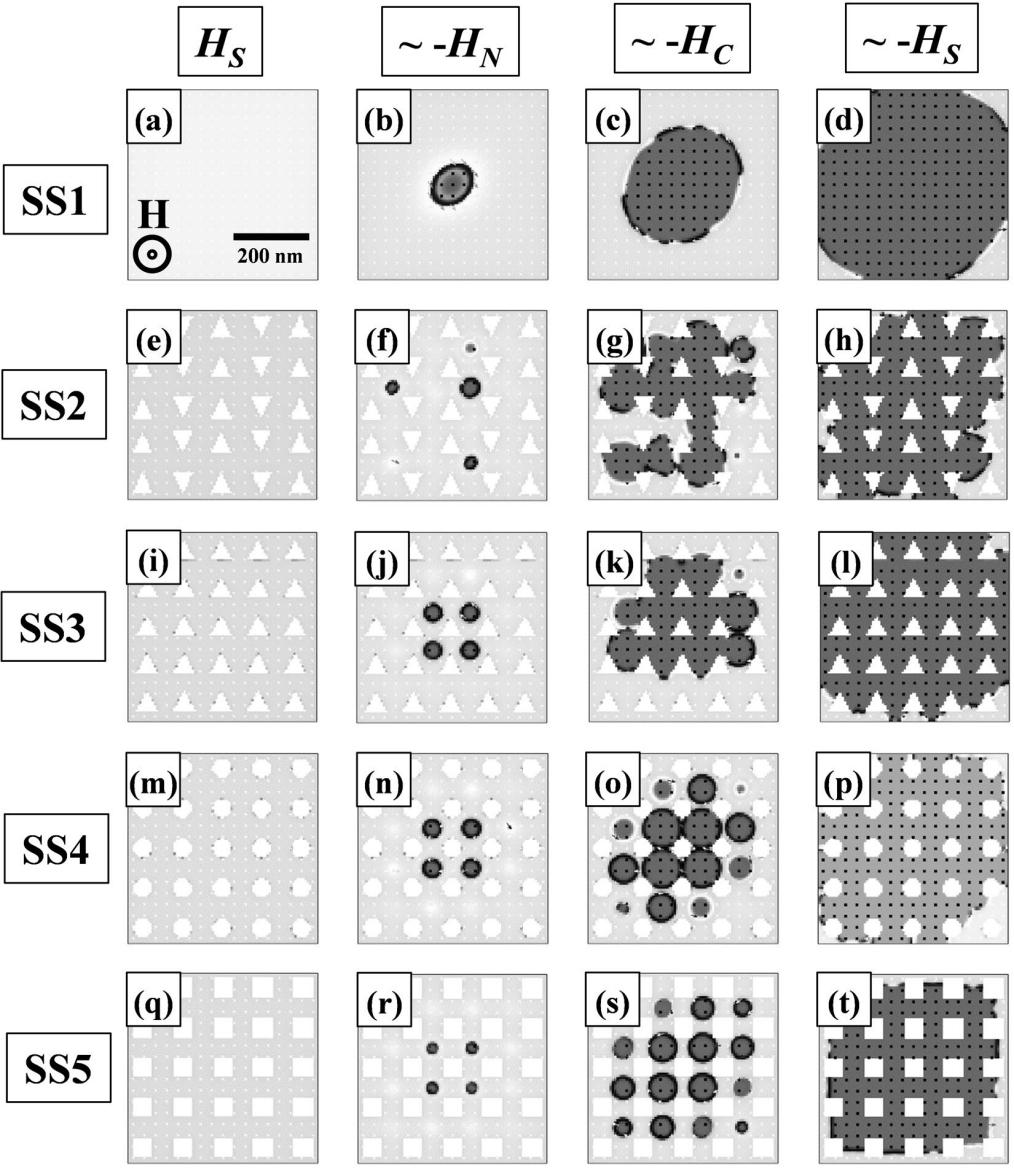
OOMMF simulated domain images for various MAL patterns (**a**–**d**) SS1, (**e**–**h**) SS2, (**i**–**l**) SS3, (**m**–**p**) SS4, (**q**–**t**) SS5 at *H*_*S*_, −*H*_*N*_, −*H*_*C*_, and ~−*H*_*S*_, respectively. The scale bar and direction of applied magnetic field are same for all the samples which are shown in (**a**).

In order to understand the domain formation and growth mechanism OOMMF simulations^27^ have been performed. The parameters considered in the simulations are as follows: saturation magnetization *M*_*s*_ = 1.1 × 10^6^ A/m, exchange stiffness constant *A* = 1 × 10^−11^ J/m, and anisotropy energy density *K* = 3 × 10^6^ J/m^3^. The anisotropy direction was set along the z-axis i.e. perpendicular to the sample plane along with a slight misalignment in x and y-axis (5%) to aid the simulation to reach its stopping criterion. Figure 4 shows the simulated domain images using OOMMF at *H*_*S*_, −*H*_*N*_, −*H*_*C*_, and ~−*H*_*S*_, for the continuous thin film (SS1): (a–d); and asymmetric triangular antidot (SS2): (e–h). It can be observed from Fig. 4(a–d) that a bubble nucleates and propagates uniformly under the influence of external magnetic field for sample SS1. Since the simulation sample is defect free, only a single bubble nucleates. However, in the experiment multiple nucleations take place (see Fig. 3(b,c)) due to inherent magnetic inhomogeneity in the thin film which act as nucleation centers for the domains. The tilt in the bubble from a perfect circle to a slightly elliptical shape occurs due to the misalignment introduced to reach the stopping criterion for simulation. This is consistent with the previous reports on such systems where the bubbles are distorted elliptically due to the orientation dependence of domain wall energy in presence of the horizontal field^26^.

Similar OOMMF simulations have been performed for the MAL arrays to elucidate the effect of periodic defects on the domain structure. However here we considered the dimension of the antidots in the nanoscale which are much smaller compared to the MAL array studied experimentally in this paper. Here we note that considering similar area in the simulation as in the experimental case is not feasible from computational point of view. Further, practical applications demand the knowledge of domain structure and magnetization reversal at nano-dimension. Due to the symmetric shape of the holes in circular, square, diamond antidots, it is not possible to arrange them in asymmetric form. Nevertheless, the triangular holes pose the opportunity of exploring the asymmetric arrangements and its role in determining the domain structure and magnetization switching mechanism. We observe that, introduction of triangular holes (sample SS2) lead to formation of multiple localized bubbles as shown in Fig. 4(f). Under the influence of the magnetic field, the bubbles expand asymmetrically and coalesce with each other to complete the reversal (Fig. 4(g,h)). The arrangement of the triangular holes (alternating pairs of straight and inverted triangles similar to the design of sample ES2) in the lattice leads to asymmetry which force the bubbles to nucleate from the edges, remain pinned between two successive triangular holes, and propagate asymmetrically. This behavior is similar to the Kerr microscopy observation for the triangular antidot sample ES2 (see Fig. 3(d–f)). Hence, we note that although the local nucleation may vary at nano-dimension, the overall domain structure remains similar for micro and nano-dimensional MAL arrays.

Further we have tried to understand the domain formation and magnetization reversal by considering a few other antidot patterns with PMA. In this regard we have performed OOMMF for three more designs which are symmetric triangular antidot (sample SS3), circular antidot (sample SS4), and square antidot (sample SS5). When symmetric arrangement of the triangular holes are considered in sample SS3, the bubbles nucleate symmetrically but propagate asymmetrically as shown in Fig. 4(i–l). This indicates that the orientation of the triangular antidots also play a role in determining the nucleation and propagation of the bubbles (see Fig. 4(e–h) and (i–l)). We note that the bubble formation and propagation is symmetric in samples SS4, and SS5 due to the symmetric shape and arrangements of circular and square antidot arrays, respectively, as shown in Fig. 4(m–t). Hence, it is concluded that by varying the MAL architecture the bubble shape, nucleation, and propagation can be engineered. From above discussion we note that the experimental results on the continuous and MAL based thin films are very well reproduced by the OOMMF simulations (Fig. 4(a–h)) and can be extended to nano-dimensions. Further our OOMMF results on other MAL designs of various shape and symmetry indicate the nature of domain nucleation and propagation in nano-dimensional antidot arrays of Co/Pt. The domain nucleation and propagation in sample SS1–SS5 can be visualized in supplementary videos 3–7, respectively.

To study the response of magnetic domains to the thermal activation energy, magnetic relaxation measurements have been performed by Kerr microscopy at room temperature. For a given sample, it was first saturated and then a reverse magnetic field was set to a sub-coercive value to relax the spins with time under constant Zeeman energy. The mean grey scale intensity of domain images is plotted with respect to its corresponding time to obtain the relaxation curve. Similar relaxation study have been previously described using single and double exponential decay behavior in case of both continuous thin films and MAL arrays with in-plane anisotropy^9–11,28^.

**Figure 5:**
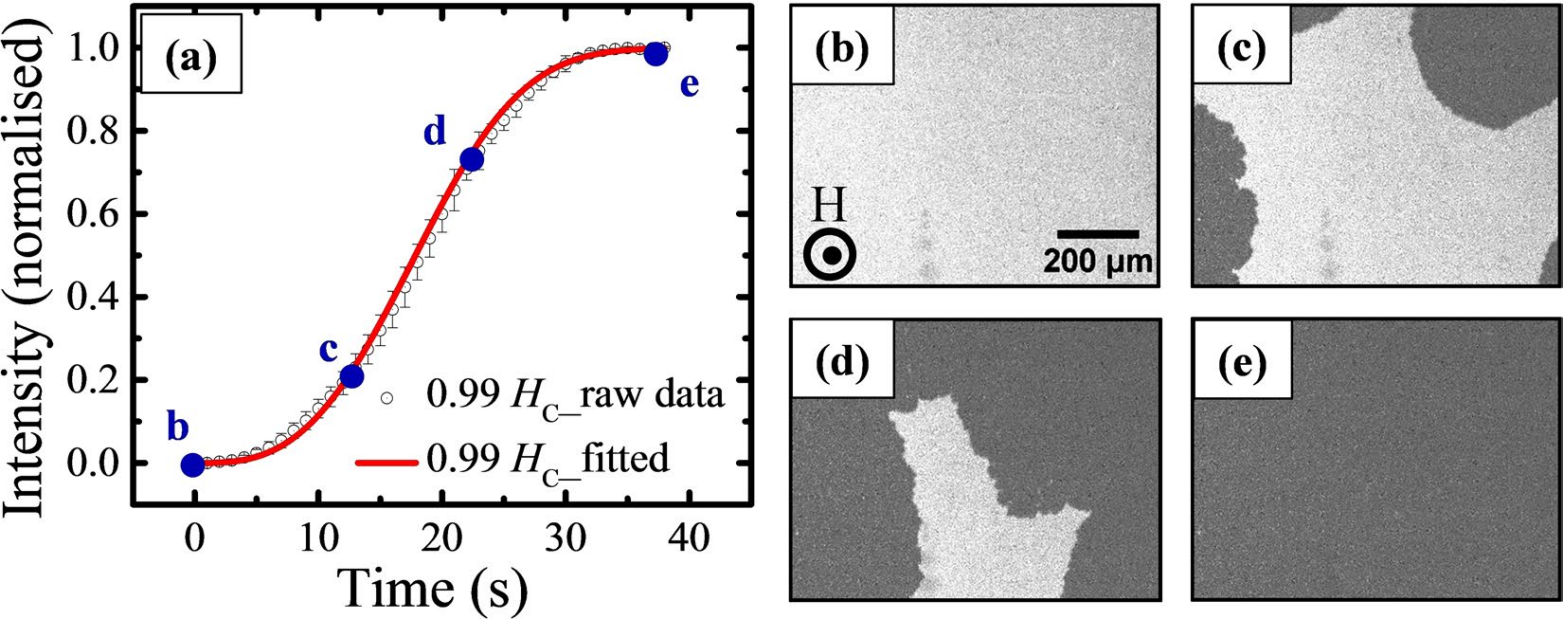
(**a**) Relaxation behavior for sample ES1 measured at *H*_*M*_ = 0.99 *H*_*C*_, where hollow black circle and red curve represent the raw data, and the fitted curve with compressed exponential function, respectively. (**b**–**e**) Show the domain images measured at 0, 13, 23, and 41 s, respectively, as marked in (**a**). The scale bar and applied field direction are same for all the images and shown in (**b**).

Figure 5(a) shows the relaxation behavior of the continuous thin film (sample ES1) at *H*_*M*_ = 0.99 *H*_*C*_ whereas (b–e) show the domain images at 0, 13, 23, and 41 seconds, respectively, as marked in (a). The image (Fig. 5(b)) at *t* = 0 s, represents the initial domain configuration for the sample just after setting the magnetic field to a fixed value. Observation of such domains with irregular, fractal boundaries in sample ES1 is associated with the domain wall response to the wide spectrum of the thermal activation energy^26^. The experimental data is fitted with the compressed exponential function^3^:

1 $$I(t)={I}_{1}+{I}_{2}(1-exp(\,-\,{(t/\tau )}^{\beta }))$$

where, *I*(*t*) is the measured Kerr intensity, *I*_1_ + *I*_2_ is the normalised Kerr intensity at saturation, *τ* is the relaxation time constant, and *β* is an exponent having a value between 1 to 3. The compressed exponential function is a generalized representation of the Fatuzzo-Labrune model^29,30^ and the former has been used to describe the relaxation in continuous thin films with PMA^31^. The exponent *β* characterizes the magnetic switching mechanism, where the low and high values of it determine whether the reversal mechanism is dominated by domain nucleation or DW motion, respectively. The fitted value of *β* for the Co/Pt thin film (sample ES1) is 3.00 ± 0.05, which reveals that the magnetization reversal in the continuous thin film is governed by DW motion.

**Figure 6:**
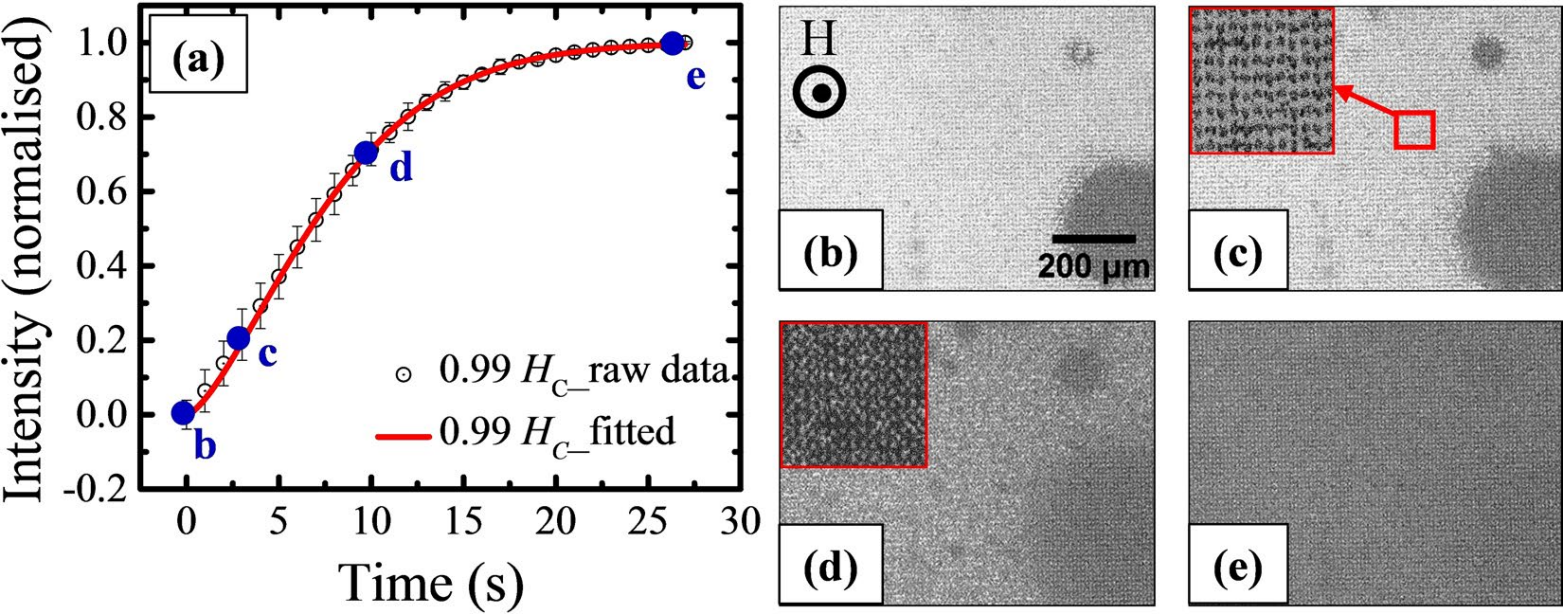
(**a**) Relaxation behavior for sample ES2 measured at *H*_*M*_ = 0.99 *H*_*C*_, where hollow black circles and red curve represent the raw data, and fitted curve with compressed exponential function, respectively. (**b**–**e**) Show the domain images at 0, 3, 10, and 27 s, respectively, as shown in (**a**). The insets of (**c)** and (**d**) are the high-resolution images of the area marked by the red box in (**c**).

Similarly, Fig. 6(a) shows the relaxation behavior for the antidot sample ES2 fitted with compressed exponential function. Figure 6(b–e) show the domain images at 0, 3, 10, and 27 s, respectively, as marked in (a). The value of *β*(1.46 ± 0.02) obtained from the best fit indicates that the switching in the antidot array is dominated by domain nucleation followed by DW motion. It is observed from Fig. 6(b) that the domain nucleation has already taken place at *t* = 0 s. This is different in comparison to the behavior in the parent continuous thin film sample ES1 because the presence of triangular holes in ES2 act as nucleation centres for the domains. The insets of Fig. 6(c,d) show the high-resolution images of the area marked by the red box in (c). It is observed that several bubbles (domains) nucleated adjacent to the antidots (holes). Then they expand and coalesce with the neighbouring bubbles to complete the reversal process.

**Figure 7:**
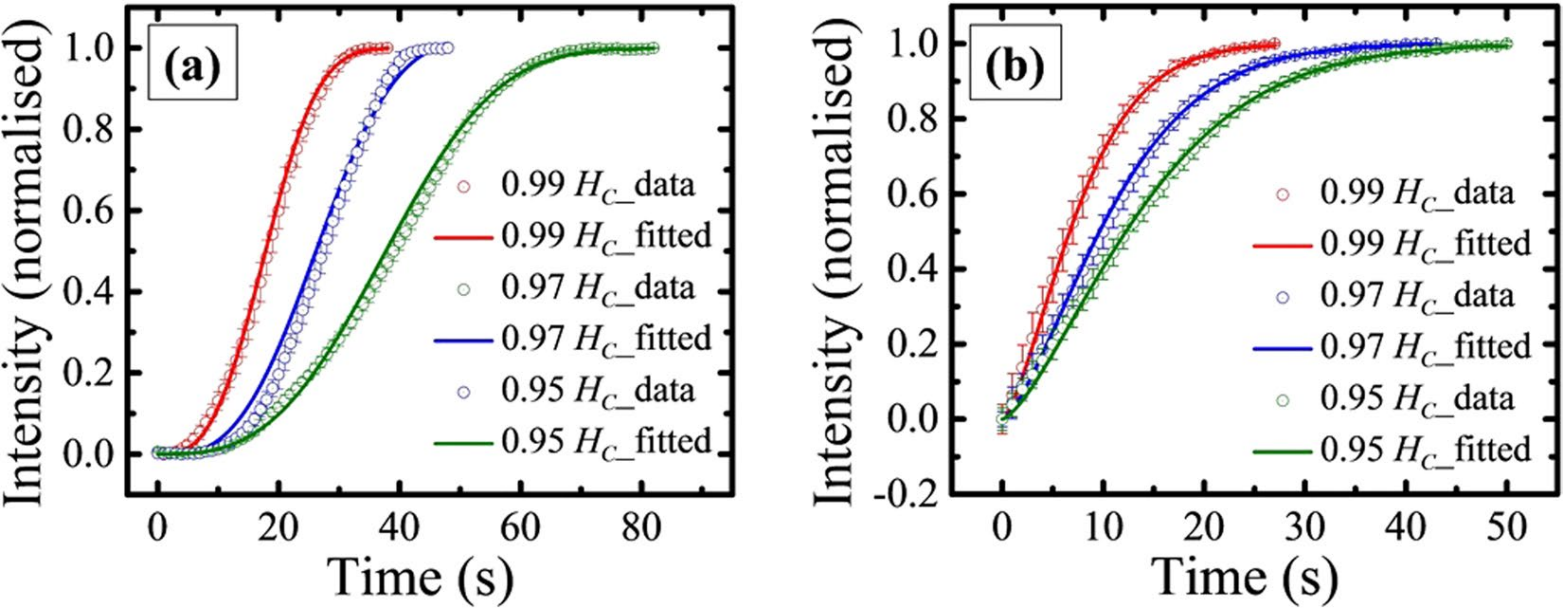
Relaxation behavior measured at *H*_*M*_ = 0.99 *H*_*C*_ (red circle), *H*_*M*_ = 0.97 *H*_*C*_ (blue circle), and *H*_*M*_ = 0.95 *H*_*C*_ (green circle) for samples ES1 (**a**) and ES2 (**b**). The solid lines represent the best fits with compressed exponential function.

In order to compare the nature and speed of relaxation for variable Zeeman energy, relaxation measurements were performed for both the continuous thin film (ES1) and triangular antidots (ES2) at different sub-coercive fields. Figure 7 shows the relaxation behavior at *H*_*M*_ = 0.99 *H*_*C*_ (red hollow circle), 0.97 *H*_*C*_ (blue hollow square), and 0.95 *H*_*C*_ (green hollow diamond), for (a) ES1, and (b) ES2. The values of *β* from the best fits for sample ES1 are 3.00 ± 0.05 (at 0.99 *H*_*C*_), 3.00 ± 0.11 (at 0.97 *H*_*C*_), 3.00 ± 0.03 (at 0.95 *H*_*C*_). For sample ES2, *β* values obtained from the best fits are 1.46 ± 0.02, 1.46 ± 0.01, and 1.44 ± 0.01 at 0.99 *H*_*C*_, 0.97 *H*_*C*_, and 0.95 *H*_*C*_, respectively. The relaxation time constant *τ* for sample ES1 are 20.16 ± 0.13 s, 29.94 ± 0.54 s, 42.64 ± 0.14 s, for 0.99 *H*_*C*_, 0.97 *H*_*C*_, and 0.95 *H*_*C*_, respectively. Similarly, *τ* for sample ES2 are 8.59 ± 0.08 s, 12.40 ± 0.10 s, 15.81 ± 0.10 s for the above fields. As expected, the relaxation becomes slow as the amplitude of the applied magnetic field is reduced. However, it should be noted that *τ* in the antidot sample ES2 is less as compared to its parent continuous thin film sample ES1. The magnetization reversal for the thin film is dominated by domain wall motion. Whereas, the reversal for the antidot sample occurs simultaneously via domain nucleation and domain wall motion. Further, the out-of-plane motion of the Bloch walls in MAL arrays with PMA face less obstruction from the periodic holes in the path of propagation. This observation is in contrary to the results obtained from thin films and antidots with similar dimension but with in-plane magnetic anisotropy where the *τ* becomes higher for the antidots^9,10^.

## Conclusion

The magnetization reversal and relaxation in perpendicular magnetic anisotropic thin films and antidots of Co/Pt have been discussed. We have observed typical bubble domains for the continuous thin film with high PMA. However by introducing periodic holes in the form of MAL arrays, it leads to the formation of multiple localized small bubbles. OOMMF simulation supports the experimental observations which is further extended to nano-dimension. In addition, the simulations reveal that the bubble nucleation and propagation are significantly affected by the structure and arrangements of the antidots. Further the nature of domains and reversal observed in the experiment (at micron scale) qualitatively matches with the simulation (at nano-scale). Hence, we note that although the domain nucleation may vary at nano-dimension, the overall domain structure remains the same for antidot arrays with perpendicular anisotropy for micro and nano-dimensional feature size of the holes.

The speed of magnetic relaxation, inferred from *τ* values, turns faster in the MAL arrays. This is in contrary to the previous reports, where the relaxation dynamics turns slower in the antidots in comparison to its parent thin films with in-plane anisotropy. Further we observe that the global relaxation is faster in out-of-plane magnetized system when compared to the in-plane ones. The possibility of achieving faster relaxation in comparison to the parent thin film in MAL arrays may have significant impact in MRAM based devices and spintronic applications where reading and writing with rapid speed is desired. The nature of magnetization reversal and domain structure in antidot arrays of Co/Pt at nano-dimension will be explored experimentally in future.

## Method

The details of the samples studied experimentally and considered in simulations are described in Table 1. The patterning for the antidots was performed on Si(100) substrate by photolithography technique (with a mask aligner manufactured by Midas System Co. Ltd., South Korea). The sample structure of ES1 and ES2 is Ta(3 nm)/Pt(3.5 nm)/Co(0.8 nm)/Pt(4.5 nm), which has been deposited on Si(100) substrate in a high vacuum chamber supplied by Mantis Deposition Ltd., UK with the base pressure better than 3 × 10^−8^ mbar. Ta layer was used to promote the (111) orientation growth of Pt to obtain high PMA. Top Pt layer is deposited to maintain the structural symmetry and avoid oxidation of the Co layer. The growth uniformity in the layers was maintained by rotating the substrate at 20 rpm during deposition. The films were prepared using DC (for Co, and Ta) and RF (for Pt) magnetron sputtering at the Ar deposition pressure of ~5 × 10^−3^ mbar. The shape, size, inter-separation between the holes of the MAL arrays were imaged using scanning electron microscopy (SEM) by Carl Zeiss AG, Germany. The study of magnetization reversal and relaxation have been performed using MOKE microscopy (in polar mode) supplied by Evico Magnetic Ltd., Germany. Micromagnetic simulations have been performed using the OOMMF package^27^ to study the domain formation in both continuous thin film and MAL arrays of different size and shapes. The simulations have been performed over an area of 500 nm × 500 nm. The cell size (0.8 nm) used in the simulation is equal to the thickness of the samples considered in the experiment.

**Table 1:** Details of experimental and simulated samples.

Sample name	Sample class	Sample type	Length of side/diameter (d)	Centre to centre distance (D)
ES1	Experimental	Continuous thin film	—	—
ES2	Experimental	Triangular antidot (assymmetric arrangemet)	~4.8 *μ*m	Horizontal ~ 9.5 *μ*m, Vertical ~ 11.9 *μ*m
SS1	Simulated	Continuous thin film	—	—
SS2	Simulated	Triangular antidot (assymmetric arrangemet)	50 nm	100 nm
SS3	Simulated	Triangular antidot (symmetric arrangemet)	50 nm	100 nm
SS4	Simulated	Circular antidot	50 nm	100 nm
SS5	Simulated	Square antidot	50 nm	100 nm

## Electronic supplementary material

Video 1

Video 2

Video 3

Video 4

Video 5

Video 6

Video 7
